# The Evaluation and Quantitation of Dihydrogen Metabolism Using Deuterium Isotope in Rats

**DOI:** 10.1371/journal.pone.0130687

**Published:** 2015-06-23

**Authors:** Radomir Hyspler, Alena Ticha, Henk Schierbeek, Alexander Galkin, Zdenek Zadak

**Affiliations:** 1 Department of Research and Development, University Hospital Hradec Kralove, Hradec Kralove, Czech Republic; 2 Division of Mother and Child, University of Amsterdam, Amsterdam, Netherlands; 3 School of Biological Sciences, Queen’s University, Belfast, United Kingdom; Texas Tech University Health Science Centers, UNITED STATES

## Abstract

**Purpose:**

Despite the significant interest in molecular hydrogen as an antioxidant in the last eight years, its quantitative metabolic parameters *in vivo* are still lacking, as is an appropriate method for determination of hydrogen effectivity in the mammalian organism under various conditions.

**Basic Procedures:**

Intraperitoneally-applied deuterium gas was used as a metabolic tracer and deuterium enrichment was determined in the body water pool. Also, *in vitro* experiments were performed using bovine heart submitochondrial particles to evaluate superoxide formation in Complex I of the respiratory chain.

**Main Findings:**

A significant oxidation of about 10% of the applied dose was found under physiological conditions in rats, proving its antioxidant properties. Hypoxia or endotoxin application did not exert any effect, whilst pure oxygen inhalation reduced deuterium oxidation. During *in vitro* experiments, a significant reduction of superoxide formation by Complex I of the respiratory chain was found under the influence of hydrogen. The possible molecular mechanisms of the beneficial effects of hydrogen are discussed, with an emphasis on the role of iron sulphur clusters in reactive oxygen species generation and on iron species-dihydrogen interaction.

**Principal Conclusions:**

According to our findings, hydrogen may be an efficient, non-toxic, highly bioavailable and low-cost antioxidant supplement for patients with pathological conditions involving ROS-induced oxidative stress.

## Introduction

Molecular hydrogen (H_2_) was for centuries considered as an physiologically inert and non-breathable gas for higher aerobic organisms including mammals. Due to its low water solubility, hydrogen was used as an inert component of breathing mixtures for deep diving and its inertness was confirmed using tritium tracer studies in 1994 [1].

In 2007, hydrogen antioxidant properties were described [2] and its ability to react directly with hydroxyl radical (OH^∙^) was proposed. Despite significant interest in molecular hydrogen as an antioxidant in the last eight years and its generally accepted efficiency [3,4,5,6], its quantitative metabolic parameters *in vivo* are still lacking. Hydrogen combines numerous advantageous properties not seen together in other antioxidants, such as high diffusion coefficient and bioavailability, non-toxicity, presence in both hydrophilic and lipophilic environments in tissues, and the ability to quickly reach mitochondria, which are one of the major sources of reactive oxygen species (ROS) [3,4]. However, the exact molecular mechanism of hydrogen antioxidant action *in vivo* is still unknown. An earlier study suggests, [2] based on fluorescent probes, that a direct reaction with hydroxyl radical is most likely, but other mechanisms were also proposed [6].

It is imperative that care be taken to avoid the explosive range of hydrogen concentration in air (4–75% v/v). Hydrogen concentrations of about 2% in breathing-gas mixtures are safe and suitable for use at atmospheric pressure, as are any solutions of hydrogen gas [5]. During saturation of hydrogen in water, a concentration of about 0.8 mmol/l may be achieved at room temperature and standard atmospheric pressure [3]. For comparison, the concentration of oxygen in water exposed to atmospheric air (21% O_2_ v/v) is about 0.25 mmol/l at room temperature or 0.2 mmol/l at 37°C [7].

To function as an antioxidant reacting directly with ROS or to affect redox centres responsible for ROS generation, the hydrogen atoms must donate their electron to oxygen radicals, whereby they become part of the H^+^ pool, which equilibrates with the large body water pool [6]. This significantly complicates the research, and necessitates the use of the stable isotope deuterium as a metabolic tracer.

The aim of this study was to quantify hydrogen metabolism in the mammalian body under various conditions using deuterium as a tracer, and to estimate its mechanism of action *in vivo*. The importance of this research derives from the large body of evidence for the role of ROS and oxidative stress in many clinically relevant scenarios, such as ischaemia/reperfusion. Despite efforts spanning many decades to find or synthesize an effective reactive oxygen species scavenger, all candidates so far have suffered from low efficiency and bioavailability, and inability to permeate cell membranes to reach subcellular organelles. Our findings prove that hydrogen may be an efficient and low-cost antioxidant supplement for patients with pathological conditions involving ROS-induced oxidative stress.

## Material and Methods

### Chemicals and animals

Sodium borodeuteride, deuterated methanol (CH_3_OD), cobalt chloride, cobalt-doped borohydride pellets, and endotoxin were obtained from Sigma-Aldrich, Bellefonte, US. Centrifugal filters were obtained from Merck Millipore, Darmstadt, Germany.

The H_2_ and CO working reference gases, quality 6.0 and 4.7 respectively, were purchased from Linde AG, Munich, Germany. Standard Light Antarctic Precipitation (SLAP), Greenland Ice Sheet Precipitation (GISP) and Standard Mean Ocean Water (SMOW2) were purchased from the International Atomic Energy Agency (IAEA, Vienna, Austria).

A high-temperature thermal conversion elemental analyzer (TC-EA) equipped with CTC autosampler (Zwillingen, CH) coupled with a Delta XP isotope ratio mass spectrometer via a Conflo-III Interface (Thermo Fisher, Bremen, Germany) and Varian Cary 4000 spectrophotometer were used. A microsensor monometer equipped with hydrogen needle sensor was obtained from Unisense A/S, Aarhus, Denmark and was calibrated according to manufacturer instructions.

The male Wistar rats (weight 230–280 g) were obtained from BioTest, Konarovice, Czech Republic. All experiments were approved by the Institutional Animal Care and Use Committee “Animal-welfare body“, established under § 15f and 15g Law No. 246/1992 “The protection of animals against cruelty” and §5 and §8 of Notice No. 419/2012 “The protection of animals for experimental purposes”, according to local law in concordance with European Union legislation (Permission No. MSMT 18939/2013-1, the name of the Institute that approved this study: Ministry of Education, Youth and Sports of the Czech Republic).

### Synthesis of deuterium gas

Deuterium gas was prepared by the reaction of deuterated borohydride and methanol CH_3_OD [8]. The rationale behind the use of methanol instead of water was to prevent the introduction of heavy water vapors into the experimental animals. The reaction was performed according to the following equation and involved catalysis by anhydrous CoCl_2_.

$${\text{NaBD}}_{4}\;+\;4{\text{ CH}}_{3}\text{OD }\to \text{ NaB}{\left({\text{OCH}}_{3}\right)}_{4}\;+\;4{\text{ D}}_{2}\;+\text{ heat}$$

The gaseous deuterium product was collected and triple washed in saline to remove any traces of borohydride and methanol aerosol or vapors prior to intraperitoneal application to the experimental animals.

### Deuterium concentration determination

The deuterium concentration was determined using a hydrogen needle sensor electrode based on the amperometric Clark electrode principle. The electrode was calibrated and operated according to manufacturer instructions. As there is a large proportional difference between hydrogen and deuterium atomic mass, a significant isotope effect was predicted. The kinetic isotope effect for reactions taking place on an anode, defined as the ratio of rate constants for the respective reactions involving the light and the heavy isotopes, was found to be 1.15. Hence, the instrument readings were compensated for this effect. Due to the resulting lower reactivity of deuterium gas compared with hydrogen, this tracer method somewhat underestimates the probable reaction rate of hydrogen oxidation *in vivo*, which would be 10–20% higher.

The determinations were performed on each whole blood sample anticoagulated with K_2_EDTA (ethylenediaminetetraacetic acid, dipotassium salt). Additionally, the tissue concentration of hydrogen was measured in peritoneum and liver tissue.

### The determination of deuterium enrichment in body water

The H_2_ and CO working reference gases were calibrated with known reference waters, i.e. Standard Light Antarctic Precipitation (SLAP) and Greenland Ice Sheet Precipitation (GISP). Working standards SMOW2, GS 47, GS 49 and HDW1 were used for calculation of each batch of analyses.

Experiments were carried out on a high-temperature thermal conversion elemental analyzer (TC-EA) coupled with a Delta XP isotope ratio mass spectrometer via a Conflo-III Interface. The IRMS was operated at an accelerating voltage of 5 kV. The ion source was held at a pressure of 3.0 x 10^−6^ Torr, and ions were generated by electron impact at 70 eV. Subsequently, two sets of faraday cup detectors monitored signals for ions at m/z 2 (^1^H/^1^H) and m/z 3 (^2^H/^1^H) for H_2_ gas, as well as m/z 28 (C^16^O) and m/z 30 (C^18^O) for CO. The ^2^H/^1^H ratios were corrected for the H_3_^+^ effect. The dynamic range of the instrument is between 0.2 and 50 V. The reactor consisted of a glassy carbon tube filled with carbon chips (IVA, Meerstadt, Germany). The following conditions were used: reactor temperature 1420°C, GC column temperature 90°C, helium flow 110 mL/min, and two reference gases, hydrogen 6.0 and carbon monoxide 4.7.

Aliquots of 0.1 μL plasma filtrate were injected by a CTC autosampler into the TC-EA/IRMS system. Samples were analyzed in the dual measurement mode. Each analytical cycle consisted of three pulses of the hydrogen reference gas introduced by the Con Flow III unit followed by measurement of the eluting hydrogen peak. After a quick swap to a different cup setting, the eluting carbon monoxide was measured, followed by three pulses of CO reference gas. Each sample was measured five times and calculated against the reference gases injected in the same run. The first two results of each data set were skipped due to carry-over items and the remaining deuterium isotope abundances of the water samples were expressed in delta per mil (δ pm) and transformed to deuterium-hydrogen ratio (tracer to tracee ratio). The ^2^H/^1^H ratios were corrected for the H_3_^+^ effect, which was determined before each sequence.

### Tracer application and experimental protocol

The animals were divided into four groups consisting of six animals (total number 24 rats) and subjected to tracer application as follows: control group–FiO_2_ in inhaled air approx. 21 kPa; inflammation group–with intraperitoneally applied endotoxin (1 mg in 500 µl) to provoke oxidative burst in leukocytes, FiO_2_ in inhaled air approx. 21 kPa; hyperoxia group–FiO_2_ in inhaled gas approx. 100 kPa; hypoxia group FiO_2_ in inhaled gas approx. 10.5 kPa. All experiments were carried out under normobaric conditions, and the conditions of inhalation mentioned above lasted for the whole tracer experiment.

The tracer experiment lasted for six hours. A dose of two milliliters of triple-washed deuterium gas was applied intraperitoneally to the experimental animals every hour (time interval 0-5h). This dose approximately equals the amount of gas absorbable by the peritoneum. Blood samples, anticoagulated with K_2_EDTA, were obtained from the retro-orbital sinus under ketamine anesthesia every two hours (time 2h, 4h, 6h) to determine the deuterium gas level and deuterium enrichment of body water. The animals were euthanized by exsanguination under deep anaesthesia (ketamine-xylazine) and the deuterium gas concentration was determined in the peritoneal cavity (iliac fossa) and liver tissue. The deuterium gas concentration was determined in whole anticoagulated blood. A second blood aliquot was centrifuged (1 500 g for 10 min) and the obtained plasma filtered by centrifugal filters to remove protein (14 000 g for 10 min). The filtrate was frozen for further deuterium enrichment analysis [9,10].

### Ability of hydrogen to scavenge superoxide or prevent its formation

The basis for the measurement is the reduction reaction of superoxide anion with acetylated cytochrome *c*. The standard preparation of bovine heart submitochondrial particles (SMP) was used [11]. Hearts from freshly slaughtered cows were collected from a local Ballymena abattoir.

Nicotinamide adenine dinucleotide (NADH)-dependent formation of superoxide radicals by Complex I was monitored at 30°C as the reduction of 27 μM acetylated cytochrome *c* at 550nm (ε = 21.5 mM^-1^×cm^-1^) in a standard mixture comprising 0.25 M sucrose, 50 mM Tris-HCl and 0.2 mM EDTA (pH = 8.6). In this assay the rate of superoxide formation is determined as the superoxide dismutase-sensitive rate of acetylated cytochrome *c* reduction measured with or without CuZn-superoxide dismutase (SOD). The reaction was initiated by addition of 50 μM NADH to 200 μg/ml of SMP in the presence of 10 μM rotenone. After the initial rates were recorded, 20 units/ml SOD was added to estimate the non-superoxide mediated fraction. It should be noted that in our experiments a significant fraction (20–25%) of the NADH-supported reduction of acetylated cytochrome *c* was insensitive to superoxide dismutase (all methodological details are in cit. 11).

To evaluate the effect of gaseous hydrogen (H_2_) on the reaction mixture (1ml total volume + ~1 ml head space), the solution containing everything except SMP and NADH was flushed with a gentle flow of H_2_ for 6 min under constant stirring prior to adding the enzyme and initiating the reaction. The hydrogen was obtained by the successive dissolving of several sodium borohydride pellets in a 25 ml wash-bottle connected by silicone tubing to an additional wash-bottle for trapping borohydride aerosol, and then to the flow-through headspace of the spectrophotometer cuvette. Three experiments in total using two different SMP preparations were carried out.

### Statistics

All statistical analyses were performed using SigmaStat software version 3.1. (Systat Software Inc., US). Data are presented as median and interquartile range in the case of animal experiments and as mean and standard deviation in the case of *in vitro* experiments. The number of animals or independent experiments is mentioned in the appropriate method description. The statistical differences between the groups were tested using Mann-Whitney Rank Sum test. For *in vitro* experiments reported values are the mean ± SD.

### Assumptions and calculations

Apart from expiration (or diffusion through the skin) of the intact molecule or oxidation to water, there are no other known metabolic fates of hydrogen or deuterium gas in the mammalian organism. This element does not enter any other metabolic reactions. All oxidized deuterium becomes part of the body water and is quickly and evenly distributed in it. The body water pool is considered stable throughout the experiment. There is no known interaction between hydrogen metabolism and metabolism of the anesthetics used.

**Fig 1:**
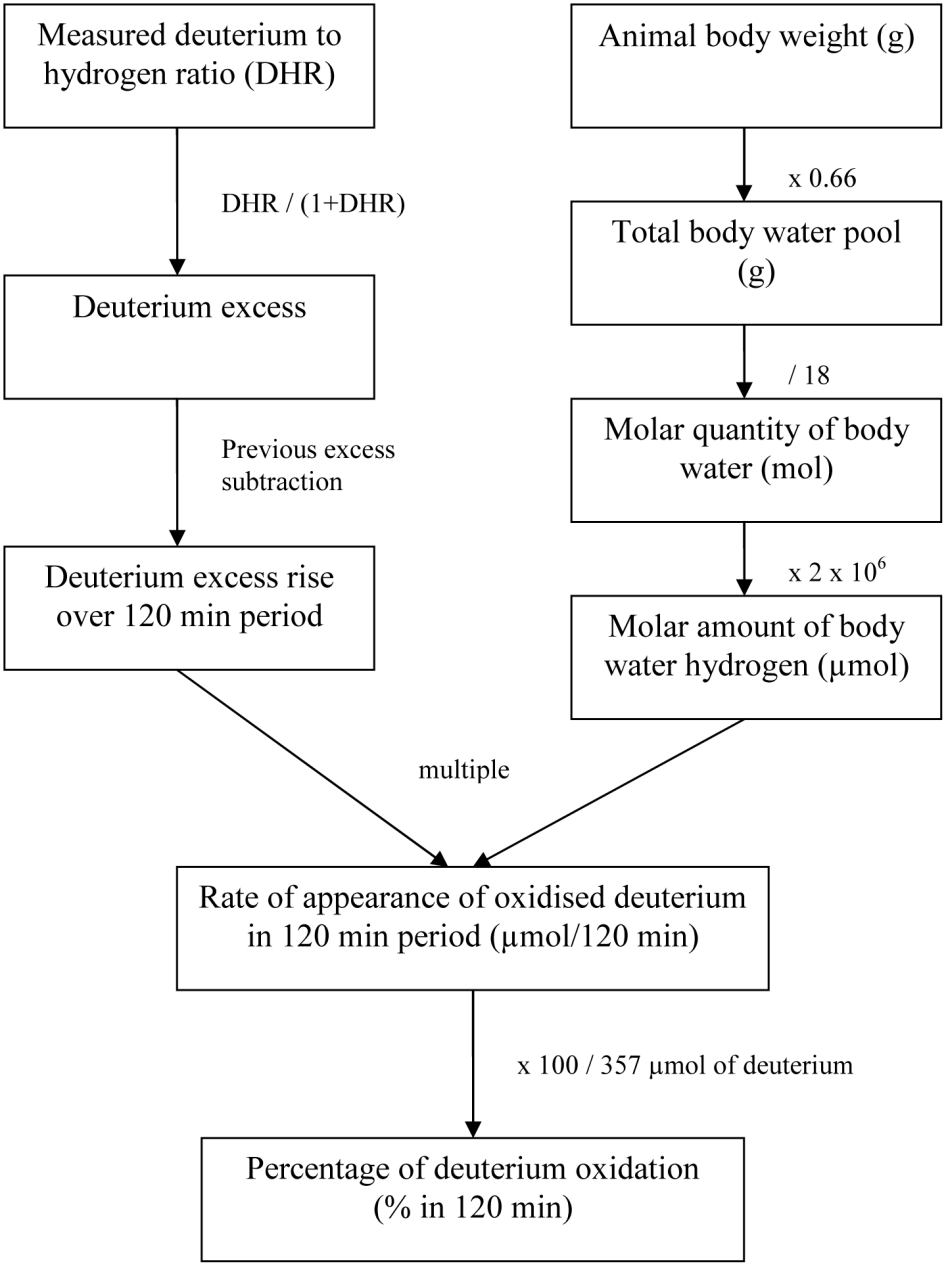
The procedure of exact metabolic calculation.

The size of the body water pool was calculated as 66% of total body weight [12]. This water weight was converted to the molar quantity of body water (dividing by the molecular weight of water). From this the molar quantity of atomic hydrogen (multiplying by two), multiplied by the measured “Isotope Excess” change between samplings, led to the “Rate of appearance” each 120 min period. The deuterium dose per two-hour period was calculated as 357 µmol of deuterium atoms. The chart of the precise steps in the calculation is presented in Fig 1.

## Results

During the tracer experiment no significant concentration of deuterium gas was found in peripheral blood. This was probably due to a large first-pass effect through the lungs, where almost all dissolved deuterium gas is eliminated by expiration. Locally, there was a high concentration in the peritoneal layer (iliac fossa, mean ± SD, 355 ± 114 µmol/l) and in the liver (244 ± 36 µmol/l).

**Fig 2:**
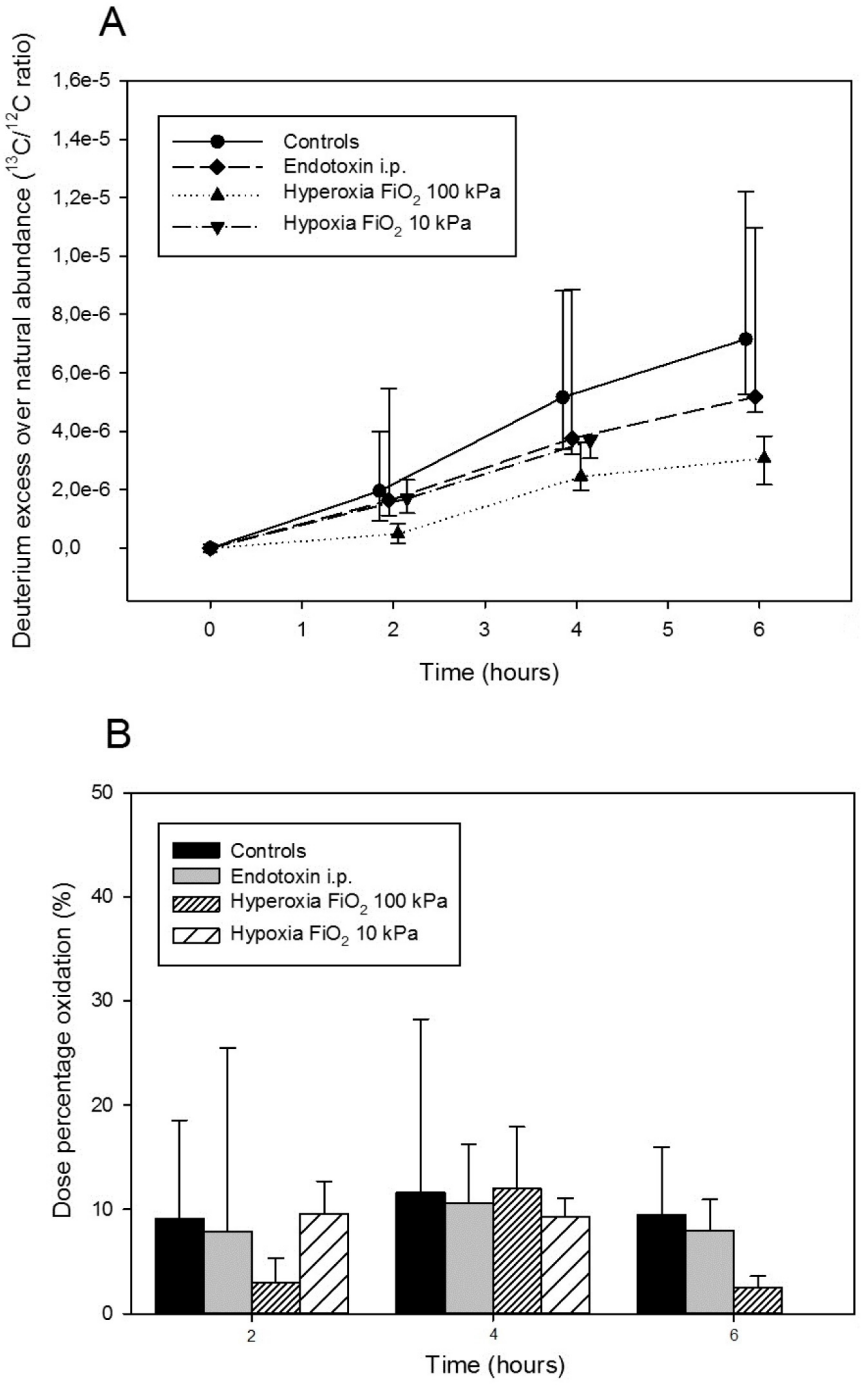
Deuterium oxidation in vivo. Deuterium isotope excess in body water from deuterium gas oxidation in vivo (A) and oxidation percentage of supplied dose of deuterium gas (B).

Significant deuterium oxidation *in vivo* was observed under the various physiological conditions. The exact time-profile of deuterium isotope excess is shown in Table 1 and Fig 2A. It shows a steady increase in deuterium enrichment of body water due to deuterium oxidation. The cumulative oxidized fraction of the supplied tracer dose of deuterium during two-hour intervals is demonstrated in Fig 2B to clarify the speed of deuterium oxidation in each interval. The highest oxidation was observed under physiological conditions and FiO_2_ 21 kPa (control group). A surprising outcome was a mild reduction in oxidation rate under the influence of endotoxin as well as under hypoxia (FiO_2_ 10.5 kPa), although it was statistically non-significant. The hypoxia experiment ended after 4 hours, by reason of death of experimental animals, probably due to the combined effects of normobaric hypoxia and repeated anesthesia causing apnea. The lowest deuterium oxidation, less than half, was observed under conditions of pure oxygen atmosphere inhalation (FiO_2_ approx. 100 kPa) lasting for the whole six hours, and was statistically significantly lower compared to the control group (p < 0.05).

**Table 1:** Isotope excess of deuterium in body water and oxidized fraction of supplied dose of deuterium gas.

Group	Time (hours)	2	4	6
Controls	Isotope excess (x10^-8^)	196 (119;337)	516(400;792)	716(597;1120)
	Oxidized fraction (%)	9.1(5.75;15.8)	11.6(8.1;20.3)	9.5(9.25;15.0)
Endotoxin	Isotope excess (x10^-8^)	163(113;435)	376(332;724)	518(482;905)
	Oxidized fraction (%)	7.9(5.45;20.3)	10.6(9.55;14.5)	7.95(5.15;10.7)
Hyperoxia	Isotope excess (x10^-8^)	49.6(24.5;74.5)*	245(198;335)*	308(222;371)*
	Oxidized fraction (%)	3.0(1.48;4.73)*	12(11.6;16.6)	2.5(1.73;3.48)*
Hypoxia	Isotope excess (x10^-8^)	170(130;231)	368(324;378)	XX
	Oxidized fraction (%)	9.6(7.15;12.7)	9.3(8.38;10.3)	XX

Data are presented as median (25th and 75th percentile)

* p< 0.05

Overall, the *in vivo* experiment suffered from a fairly large scatter in the oxidation rate of individual animals. This is due to a variety of reasons: extreme tracer volatility, interindividual differences in deuterium absorption from the peritoneal cavity, diverse blood circulation parameters as well as differences in respiratory elimination of deuterium from the animals. Undoubtedly, much more stable concentrations of deuterium in the body tissues could be obtained by a 2% deuterium-air (v/v) inhalation mixture, but this mixture would be very costly and difficult to obtain.

**Fig 3:**
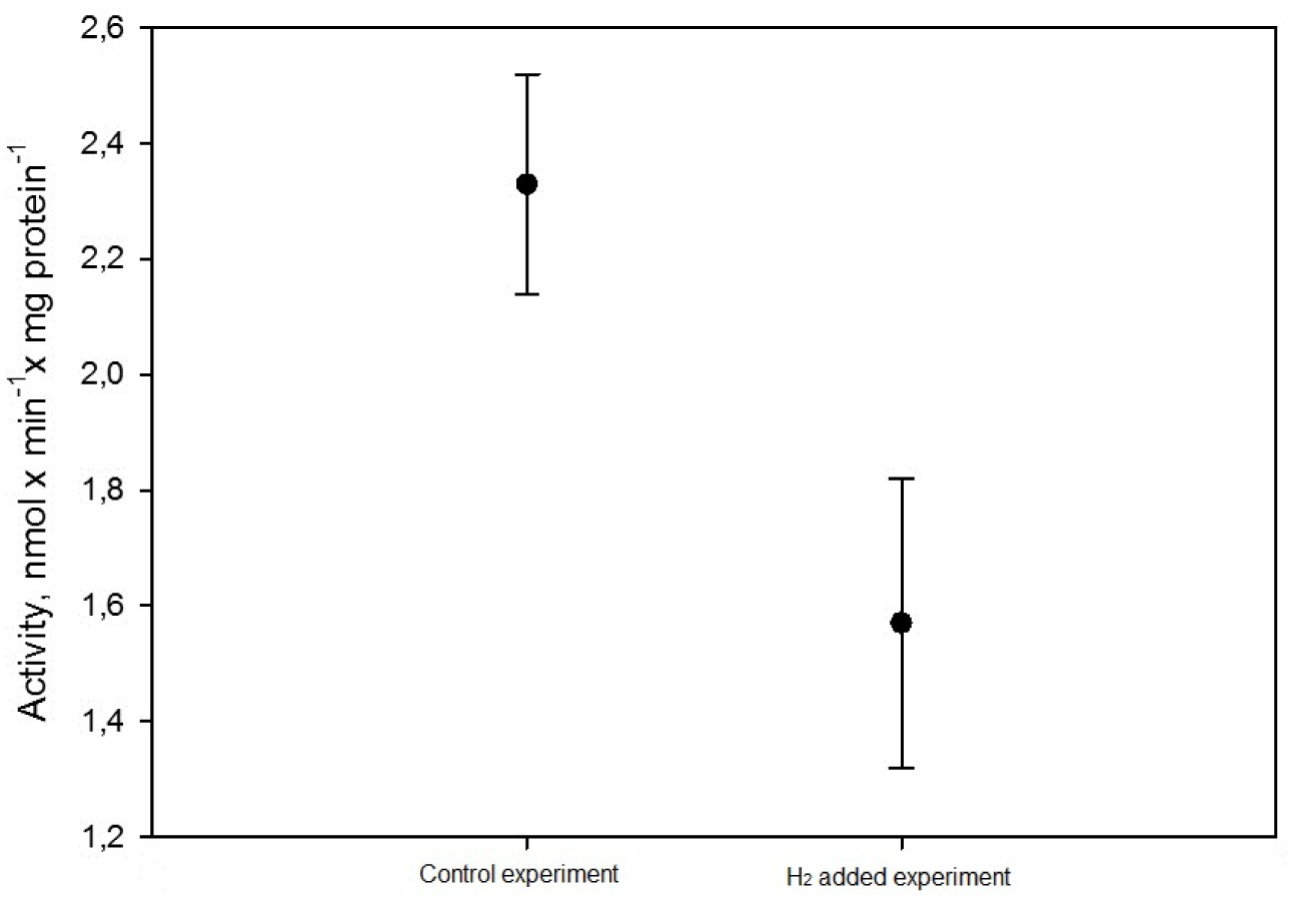
Effect of gaseous hydrogen on the production of superoxide anion by complex I (significant reduction, *p* = 0.0002).

Mitochondria are usually considered as one of the major sources of ROS. Therefore, we decided to test whether hydrogen affects mitochondrial superoxide production using preparation of isolated SMP. During *in vitro* experiment, a significant reduction of superoxide formation by mitochondrial complex I was found to be induced by the presence of hydrogen. Production of superoxide anion by complex I in SMP was 2.33±0.19 nmol×min^-1^×mg^-1^, while in the presence of hydrogen a significant (*p* = 0.0002) reduction to 1.57±0.25 nmol×min^-1^×mg^-1^ was found (Fig 3).

## Discussion

The availability of exceedingly more sensitive instrumentation and superior analytical methods allowed us to detect and quantify the oxidation rate of hydrogen *in vivo*. *Given* hydrogen’s known significant physiological impact, this study clarifies an important issue. The method used in the pioneering work on hydrogen metabolism in mammals which declared hydrogen as a physiologically inert gas [1] suffered from a relatively high detection limit for tritium tissue incorporation of about 100 nmol H_2_×g^-1^×min^-1^, which is two orders of magnitude higher than values found in our study.

The absence of measurable concentrations of deuterium in systemic venous blood is explained by the first-pass elimination effect in the lungs which is similar for all gases [13]. Intraperitoneal administration of hydrogen tracer prevents its rapid expiration and ensures a sufficient and continuous supply of this highly volatile molecule to the organism.

The measurable oxidation of stable hydrogen isotope was observed in rats, confirming its antioxidant properties *in vivo*. Despite a large first-pass effect in the lungs, a significant proportion of the supplied tracer dose (approx. 10%) was oxidized to water. This is in concordance with other substances such as uric acid, a well known antioxidant of the human body, which is oxidized by ROS to allantoin to the extent of 3–5% before excretion [14].

The ratio of oxidized to expired deuterium varies in response to various physiological conditions, although deuterium oxidation was not statistically different in the hypoxia and inflammation groups relative to controls. Most surprisingly, pure oxygen inhalation known to raise ROS formation [15], significantly decreased deuterium oxidation, rather than increase it. Also, hydrogen is known to be stable to oxidation in water solutions in the absence of a catalyst, such as transition metal [6]. The most abundant transition metal ion *in vivo* is iron, which does take part in ROS generation (Fenton and Haber-Weiss reactions). Superoxide anion (and subsequently other ROS) can be generated spontaneously from oxidation of ferrous to ferric ion [7].

These recent opinions emphasize the roles of perferryl and dominantly ferryl radicals, which are iron-oxygen complexes able to either produce ROS or to react directly with biological molecules (*i*.*e*. lipids). *In vivo*, the ferryl species FeO^2+^ could be the exact target of hydrogen gas antioxidant action Fe = O^2+^ + H_2_ → Fe^2+^ + H_2_O. Thus, in the case of pure oxygen inhalation, the ROS intracellular levels are elevated, but the ferryl species are scavenged by the reaction Fe = O^2+^ + H_2_O_2_ → Fe^3+^ + H_2_O + O_2_^·-^ [7] and diminished hydrogen oxidation results. Also, the increase in oxygen partial pressure in blood is known to decrease hemoglobin autoxidation (superoxide and methemoglobin formation) due to the much lower fraction of deoxyhemoglobin available for interaction with molecular oxygen [16]. The central role of iron and its electron transfer ability in hydrogen oxidation *in vivo* may be hypothesized, although it must be remembered that there are many other compounds or atoms which interact with iron atoms (sulfhydryl groups of glutathione, amino acid residues in proteins, heme in hemoproteins or sulfur atoms in iron-sulfur clusters) [16]. This mechanism is not in contradiction to the pioneering experiments of the Ohsawa group [2], as they found lower cytotoxicity of the hydroxyl radical in the presence of hydrogen in cell cultures, and proposed that the two species react. The more probable explanation according to our experiments is the prevention of hydroxyl radical formation rather than its scavenging.

In the case of hypoxia, low hydrogen oxidation is caused by lower ferryl species formation. The negligible effect of endotoxin intraperitoneal injection may be explained by the low effect of oxidative burst of white blood cells or by the lack of direct reaction of ROS (namely hydroxyl radical) with hydrogen, and the lower levels of ferryl or perferryl species.

Although the beneficial effect of hydrogen is generally accepted, the mechanism is not clearly understood. It has been suggested that the scavenging action of gaseous hydrogen is due to the reaction with hydroxyl radical *in vitro* [17] and in cell cultures [2]. However, metal ions such as iron-sulphur centres (FeS) may be the site where a hydrogen molecule may interact with metalloproteins, by analogy with H_2_-dependent hydrogenases. There are eight FeS clusters in mitochondrial complex I and in routine steady-state respiration they are fully reduced. In this form they are potential donors for one-electron reduction of molecular oxygen and formation of superoxide. Therefore, direct transfer of an electron to a H_2_ molecule from the reduced FeS centres followed by fast oxidation of hydride to water is possible. In addition, bimolecular reaction between molecular oxygen and a weakly-bound complex between ferrous iron and water is an accepted mechanism for production of superoxide [16]. Therefore, a decrease of ROS production can be explained as a direct reaction with the complex via replacement of a water molecule by a hydrogen molecule. There is experimental evidence of the superoxide-scavenging properties of hydrogen in tissues.[18].

## Conclusions

The performed experiments proved the antioxidant properties of hydrogen *in vivo* at a molecular level. Data presented here strongly suggest that gaseous hydrogen can play a beneficial role as an antioxidant. The mechanism probably differs from the widely-proposed direct hydrogen reactivity with hydroxyl radicals and deserves further research. Our study supports the use of hydrogen as a valuable therapeutic option for its non-toxic properties, rapid effect and low cost. It may be safely applied to human patients as a component of an inhalation mixture and may find use in numerous clinical conditions where the elevated level of ROS plays a significant role.
